# Infection patterns of dengue, Zika and endosymbiont *Wolbachia* in the mosquito *Aedes albopictus* in Hong Kong

**DOI:** 10.1186/s13071-020-04231-x

**Published:** 2020-07-20

**Authors:** Elaine Y. Y. Huang, Annette Y. P. Wong, Ivy H. T. Lee, Zhe Qu, Ho Yin Yip, Chi-wah Leung, Shuk-may Yin, Jerome H. L. Hui

**Affiliations:** 1 School of Life Sciences, Simon F.S. Li Marine Science Laboratory, Key Laboratory of Agrobiotechnology, The Chinese University of Hong Kong, Hong Kong, China; 2 Pest Control Advisory Section, Food and Environmental Hygiene Department, The Government of the Hong Kong Special Administrative Region (HKSAR), Hong Kong, China

**Keywords:** *Aedes*, Dengue, Mosquito, *Wolbachia*, Zika

## Abstract

**Background:**

The mosquito *Aedes albopictus* is a vector of dengue and Zika viruses. Insecticide-resistant mosquito populations have evolved in recent decades, suggesting that new control strategies are needed. Hong Kong has a monsoon-influenced humid subtropical climate, which favours the spread of mosquitoes. However, baseline information on the composition and dynamics of the occurrence of endosymbiont *Wolbachia* in local *Ae. albopictus* is lacking, hindering the development of scientifically-informed control measures. This study identifies the presence and absence of dengue and Zika viruses, and *Wolbachia* infection in *Aedes albopictus* in Hong Kong.

**Methods:**

Oviposition traps were set at 57 areas in Hong Kong, and both immature and adult mosquitoes were collected on a monthly basis between April 2018 and April 2019 as the study sample. Each individual mosquito in this sample was processed and screened for the presence of the dengue and Zika viruses and the endosymbionts *Wolbachia w*AlbA and *w*AlbB with PCR.

**Results:**

Totals of 967 and 984 mosquitoes were tested respectively for the presence of dengue and Zika viruses, and no trace of either infection was found in these samples. The presence of *w*AlbA and *w*AlbB was also tested in 1582 individuals. Over 80% of these individuals were found to be stably infected with *Wolbachia* throughout the thirteen-month collection period (~ 47% singly-infected; ~ 36.8% doubly infected with both *w*AlbA and *w*AlbB).

**Conclusions:**

The high degree of *Wolbachia w*AlbA and *w*AlbB infection in *Ae. albopictus* mosquitoes in Hong Kong, coupled with the absence of any signs of infection by dengue and Zika viruses, contrasts significantly with the pattern of mosquito infection in other parts of Asia. Further studies of the infection pattern in local mosquitoes are warranted before mosquito control strategies used in other regions are implemented in Hong Kong.
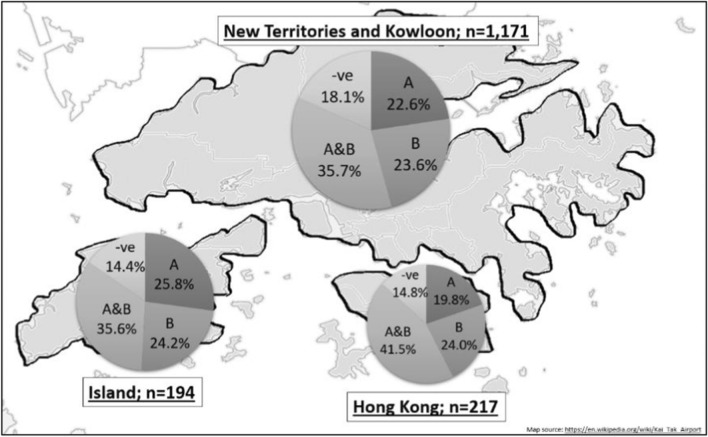

## Background

The Asian tiger mosquito (*Aedes albopictus*), an insect pest also known from its habits as the forest day mosquito, which used to be a species confined to Southeast Asia. In recent years, however, it has spread to many other parts of the world, and can now be found in Africa, America, Europe and the Middle East. *Aedes albopictus* males primarily feed on nectar, while females typically feed on the blood of birds and mammals, including humans. The female *Ae. albopictus* often feeds by biting multiple hosts [1, 2]. *Aedes albopictus* is therefore an ideal channel for the transmission of vector-borne diseases, including dengue and Zika viruses.

Dengue virus is a single positive-stranded RNA virus of the family Flaviviridae, and can cause dengue fever (including breakbone fever, dandy fever, dengue hemorrhagic fever and dengue shock syndrome) in humans after transmission by the *Aedes* mosquito. The mortality rate of untreated dengue shock syndrome is more than 20%, and the reported incidence of dengue fever has increased 30-fold over the past half century, resulting in 22,000 deaths annually [3]. According to current estimates, there are ~ 390 million dengue infections per year [4], and 3.9 billion people in 128 countries are at risk of infection [5]. In Hong Kong, the first diagnosis of dengue hemorrhagic fever was reported in 1984 [6], and it has been a statutory notifiable disease in Hong Kong since 1994 [7]. Locally acquired dengue fever has also been reported, with the first confirmed case occurring in 2003 [8]. Between 1994 and 2008 there were a total of 358 notifications, and annual numbers of notifications now range from single to double digits [7]. As modern travellers frequently move between countries, a number of cases brought in by travellers from endemic areas have also been confirmed [9–11]. The number of dengue cases reported in Hong Kong usually correlates with the occurrence of outbreaks in other Southeast Asian countries, such as those reported in 2007 or the more recent outbreak in Guangdong Province which affected 40,000 people in 2014.

The Zika virus is another single positive-stranded RNA virus of the family Flaviviridae, which can be transmitted to humans by the *Aedes* mosquito. Its symptoms include conjunctivitis, fever, rash, and muscle and joint pain. Most people infected by the Zika virus do not develop symptoms. Even if they do, these are generally mild. Nevertheless, Zika virus infection during pregnancy can cause microcephaly and other congenital malformations in infants, and in some cases preterm births and miscarriages. The largest documented Zika virus outbreak occurred in 2013 in French Polynesia, where 8200 cases of infection were reported in a total population of 268,000 people [12]. A total of 5168 cases associated with symptoms of the Zika virus were reported in the USA in 2016, but this figure had fallen to a mere 72 cases in the mainland USA and 148 cases in its overseas territories (Samoa, Virgin Islands, Puerto Rico, Guam and the Northern Marianas) in 2018 (Centers for Disease Control and Prevention 2019) [13]. It is not clear whether this marked decrease in the incidence of the Zika virus indicates that it is being brought under control globally, or merely in the USA and its dependencies.

Insecticides have been extensively used to control mosquito populations in recent decades, but resistance to the four main classes of neurotoxic insecticides (carbamates, organochlorines, organophosphates and pyrethroids) has gradually built up in *Aedes* mosquitoes in North and South America, Africa and Asia [14]. A new control strategy is presently under development, which features the use of the intracellular endosymbiotic Alphaproteobacterium *Wolbachia* [15]. In general, *Wolbachia* is prevalent in ~ 40% of arthropod species, and the supergroups “A” (*w*AlbA) and “B” (*w*AlbB) are known to exist in mosquitoes [16, 17]. The *Wolbachia* strains *w*AlbA and *w*AlbB from each supergroup can be naturally found in *Ae. albopictus* [18, 19]. For reasons not entirely clear, the sequences of the *16S* rRNA, *wsp* and *ftsZ* genes of *w*AlbA and *w*AlbB isolated from many parts of the world are identical, suggesting a potential lack of sequence diversity [20–22]. The *w*AlbA and *w*AlbB strains are maternally inherited, and can increase the lifespan of female mosquitoes and the period during which they produce eggs [23–25]. On the other hand, when *Ae. albopictus* males are doubly-infected with *w*AlbA and *w*AlbB, high levels of cytoplasmic incompatibility (CI) can result in crosses with uninfected or single infected females, and the removal of the two strains has no effect on lifespans or mating rates and sperm capacity [23, 26, 27]. In the *Ae. albopictus* cell line Aa23, sub-lethal doses of antibiotic treatment have revealed a strong negative correlation between the *Wolbachia* strain and dengue virus [28].

Studies of the relationship between the transmission of *Wolbachia* and dengue virus in *Ae. Albopictus* have reached different conclusions. In a head-squash assay experiment, dengue virus inhibition did not occur in *Wolbachia-*mediated *Ae. albopictus* [29]. Nevertheless, a study which measured the viral loads and *Wolbachia* densities in different organs of *Ae. albopictus* where dengue virus replication took place after ingestion concluded that *Wolbachia* does not affect replication of dengue virus, but is able to reduce viral infection of the salivary glands and transmission [30]. Furthermore, in a study of transient infection with the *Wolbachia* strain *w*Mel (from the fruit fly *Drosophila melanogaster)* in *Ae. albopictus*, transmission of dengue virus was inhibited, but no significant effects on fecundity were observed [31]. The introduction of another strain of *Wolbachia* (e.g. *w*Pip from the mosquito *Culex pipiens*) into *Ae. albopictus* was also able to induce CI [32], and this finding has also been explored as offering an alternative to the traditional strategy of releasing sterile male mosquitoes. Understanding the composition and dynamics of occurrence of *Wolbachia* in local *Ae. albopictus* is therefore of fundamental importance in providing baseline information for the development of appropriate strategies to control mosquito-borne infections. Such information is currently lacking in Hong Kong, even though *Ae. albopictus* is particularly active during the territory’s subtropical summers and cases of dengue fever have been reported from time to time. Our study aimed to gather more information on the presence of dengue and Zika viruses and *Wolbachia w*AlbA and *w*AlbB in *Ae. albopictus* at various locations in Hong Kong during a single typical year.

## Methods

### Study area and mosquito collection

To collect our samples, we used oviposition traps set in 57 areas (covering 18 districts) in Hong Kong by the HKSAR Government’s Food and Environmental Hygiene Department (FEHD) to monitor the breeding habits of the *Aedes albopictus* mosquito. More than 55 oviposition traps (ovitraps) were set in each surveyed area. An ovitrap is a 10 cm tall black plastic container with diameters measured from 5 cm (base) to 6.5 cm (top). A brownish wooden paddle is placed inside each ovitrap, and a black lid with 4 round-openings is used as cover. Approximately 170 ml dechlorinated tap water is contained in each ovitrap. The ovitraps were installed in outdoor locations for two consecutive weeks each month and inspected weekly. They were collected and replaced at regular intervals with fresh ovitraps in the same locations, to enable permanent surveillance to be maintained. The retrieved ovitraps were immediately checked for the presence of larvae. If eggs were present, they were kept in an FEHD laboratory for a week at room temperature to allow them to incubate and hatch into larvae. The mosquito larvae were examined under a microscope, and their species were identified. *Aedes albopictus* individuals, both immature and adult, were collected from 57 different outdoor locations in Hong Kong between April 2018 and April 2019 for the purpose of DNA and RNA extraction. Individuals collected from areas with an ovitrap index (= number of *Aedes*-positive ovitraps/total number of ovitraps retrieved from a particular area × 100%) above 10% were reared to adulthood at room temperature for 3 weeks in dechlorinated tap water using a mosquito breeder (BioQuip, California, USA). Aquarium fish feeds were used as a larval diet. Adults were harvested from the mosquito breeder every day and transferred to TRIzol reagent (Ambion, Texas, USA) at − 80 °C for further analysis of the presence of the Zika virus. Individuals collected from areas with an ovitrap index below 10%, along with those which had not yet reached adulthood at the end of the 3-week-incubation period, were transferred to absolute ethanol at room temperature for further analysis of potential *Wolbachia* infection.

### RNA extraction

RNA from 967 and 984 samples for dengue and Zika virus tests respectively was extracted using TRIzol reagent (Ambion), in accordance with the manufacturer’s instructions. Both gel electrophoresis and a NanoDrop measurement were employed for quality and quantity checks, to confirm the integrity and amount of the RNA extracted.

### Dengue virus test

Extracted RNA from individual samples was reverse transcribed into cDNA using the iScript™ cDNA Synthesis Kit (Bio-rad, California, USA), in accordance with the manufacturer’s instructions. This involved 300–1500 ng of RNA template, a 2 µl 5× reaction mix and 0.5 µl reverse transcriptase. Polymerase chain reaction (PCR) detection was carried out in accordance with a procedure described in previous studies, using dengue specific primers (DenF: 5′-TCA ATA TGC TGA AAC GCG CGA GAA ACC G-3′; DenR: 5′-TTG CAC CAA CAG TCA ATG TCT TCA GGT TC-3′) [33–35]. Each reaction mix includes 2 µl cDNA template, 1× PCR buffer, 0.8 mM of dNTPs, 1.5 mM of MgCl_2_, 0.4 µM of each forward and reverse primer, 11.2 µl dd H2O and 1 unit of *Taq* DNA polymerase, with the following parameters: 1 cycle of 3 min at 95 °C, 40 cycles of 30 s at 95 °C, 30 s at 55 °C and 35 s at 72 °C, and a final extension step at 72 °C for 5 mins. The amplified PCR products were examined using gel electrophoresis.

### Zika virus test

Extracted RNA was also amplified into cDNA by the same process described already. The 91-bp Zika virus isolate 1_0016_PF polyprotein gene region was targeted and amplified with the specific primers Zika4481 (5′-CTG TGG CAT GAA CCC AAT AG-3′) and Zika4552c (5′-ATC CCA TAG AGC ACC ACT CC-3′). A total volume of 15 µl was set for each reaction, including 0.3 µM for each primer, 5 µl cDNA template, 7.5 µl master mix (iTaq Universal SYBR Green Supermix) and 1.5 µl dd H_2_O. Amplification was performed on a real-time PCR machine (CFX96; Bio-rad). In addition, a set of Zika standards with known concentrations was prepared to develop a standard amplification curve each time. A negative control was set by replacing the cDNA template with water. The quantification cycle (Cq value) was set at 0.00001 pg/µl. The thermal cycling conditions were: (i) 1 cycle of 95 °C for 3 min; (ii) 39 cycles of 95 °C for 10 s, followed by 55 °C for 10 s and 72 °C for 15 s; and (iii) a final extension step at 72 °C for 15 s. Three technical replicates were conducted for each sample.

### DNA extraction

DNA from 1582 samples of *Wolbachia* was extracted using the Purelink Genomic DNA Mini Kit (Invitrogen, California, USA), with slight modifications to the manufacturer’s instructions. Individual larval or adult mosquitoes were homogenized in digestion buffer with proteinase K and incubated at 55 °C. After digestion, samples were centrifuged at 14,000× *rpm* for 3 min, and the supernatant was then transferred for further DNA extraction. To remove any RNA and isolate DNA effectively, RNase A was also added and incubated for 2 min at 37 °C. The quality and integrity of the extracted DNA was determined by gel electrophoresis and observed with the Gel Doc™ EZ imager (Bio-rad).

### Wolbachia wAlbA and wAlbB tests

PCR was used to amplify the targeted DNA fragment of *w*AlbA and *w*AlbB. Amplification was carried out on a T100™ thermocycler (Bio-rad) with the following parameters: 1 cycle of 3 min at 95 °C; 40 cycles of 30 s at 95 °C, 30 s at 56 °C and 35 s at 72 °C; and a final extension step at 72 °C for 5 min. The final volume of each reaction was 20 µl, including 2 µl DNA sample, 1× buffer, 0.8 mM of dNTPs, 1.5 mM of MgCl_2_, 0.4 µM of each forward and reverse primer, 11.2 µl dd H_2_O and 1 unit of *Taq* DNA polymerase. The forward primers used for amplifying the *w*AlbA and *w*AlbB DNA fragments were (5′-CCA GCA GAT ACT ATT GCG AAC AGT T-3′) and (5′-AAG GAA CCG AAG TTC ATG ATC CT-3′), respectively; together with a common reverse primer (5′-AAA AAT TAA ACG CTA CTC CAG CTT CTG C-3′) used in respective PCR reactions [19]. Reactions containing only water instead of DNA samples were set as negative controls. The amplified DNA fragments of *w*AlbA and *w*AlbB were 379 bp and 501 bp, respectively, and their presence was checked under gel electrophoresis.

Whenever a sample showed negative results (i.e. indicating the probable absence of *Wolbachia* infection), another PCR amplification on the *12S* rDNA gene was conducted using primers 12SAI (5′-AAA CTA GGA TTA GAT ACC CTA TTA T-3′) and 12SBI (5′-AAG AGC GAC GGG CGA TGT GT-3′) [36, 37] to retest the quality of DNA extraction. Samples which failed to amplify the *12S* rDNA gene were excluded from the data analyses. The PCR amplification process was performed much as in the previous procedure, but with slight modifications: 1 cycle of 3 min at 95 °C; 10 cycles of 30 s at 95 °C, 30 s at 50 °C and 35 s at 72 °C; 25 cycles of 30 s at 95 °C, 30 s at 59 °C and 35 s at 72 °C; and a final extension step at 72 °C at 5 min. As in the previous procedure, the amplification of *12S* rDNA gene fragments was determined by gel electrophoresis.

### Statistical tests

A Chi-square test was performed to determine whether there were significant differences between the frequency of infection in Hong Kong’s eighteen separate districts. Results were considered for the *P* values < 0.05, < 0.01, and < 0.001.

## Results

### Distribution of samples collected

**Fig. 1:**
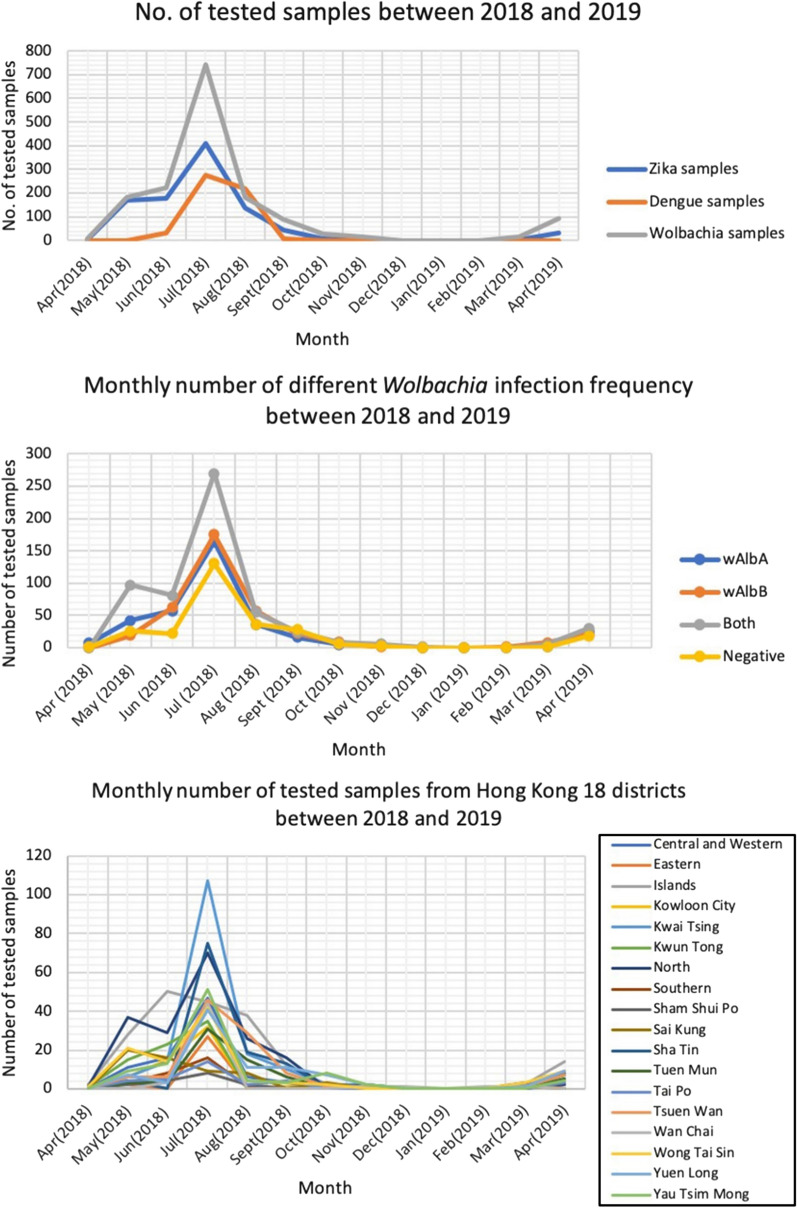
Graphs showing the number of individual mosquitoes tested for the dengue and Zika viruses and for *Wolbachia* (upper panel); *Wolbachia* infection frequency (middle panel), and tested samples from different districts (lower panel) between 2018 and 2019

A breakdown of the numbers of tested samples collected from different locations in Hong Kong during the period of April 2018 to April 2019 is given in Fig. 1. Almost half of the samples were obtained in July 2018, at the peak of the breeding season for local mosquitoes. There was a significant drop in the number of samples collected in August 2018, probably because additional mosquito controls were deployed in that month to tackle a local outbreak of dengue fever in Hong Kong. Detailed information on the samples collected in different areas in Hong Kong is given in Additional file 1: Tables S1–S8.

### Dengue virus and Zika virus tests

The 967 and 984 mosquito samples tested respectively for the presence of the dengue and Zika viruses showed no evidence of the presence of either virus. None of the samples tested contained more than 0.00001 pg/µl, indicating that both viruses were absent.

### *Wolbachia* wAlbA and wAlbB tests

Figure 1 and Table 1 illustrate the number of mosquito samples exhibiting respectively the presence of *w*AlbA (A), the presence of *w*AlbB (B), the presence of both *w*AlbA and *w*AlbB (A & B), and the absence of both *w*AlbA and *w*AlbB (negative). Forty-one samples with DNA degradation which tested negative upon *12S* rDNA amplification were excluded from the analyses. Over 80% of the mosquito samples tested were found to be *Wolbachia*-infected. 36.8% of the samples were doubly infected with both *w*AlbA and *w*AlbB. 23% were singly infected with *w*AlbA, and 24% with *w*AlbB. Detailed month-to-month results are shown in Additional file 2: Figures S1–S12.

**Table 1:** Number of individual *Ae. albopictus* mosquitoes found to be infected with *Wolbachia wAlbA* and *w*AlbB strains during the study period (April 2018 to April 2019)

Year	Month	wlbA	wlbB	wlbA & wlbB	Negative	Total
2018	April	7	0	0	1	8
	May	42	19	97	26	184
	June	57	62	81	22	222
	July	164	175	270	131	740
	August	36	57	55	36	184
	September	16	22	24	28	90
	October	5	9	8	6	28
	November	4	1	6	3	14
	December	0	0	1	0	1
2019	January	0	0	0	0	0
	February	0	1	0	0	1
	March	2	8	5	1	16
	April	25	21	30	18	94
Total		358 (22.5%)	375 (23.5%)	577 (36.8%)	272 (17.2%)	1582

**Fig. 2:**
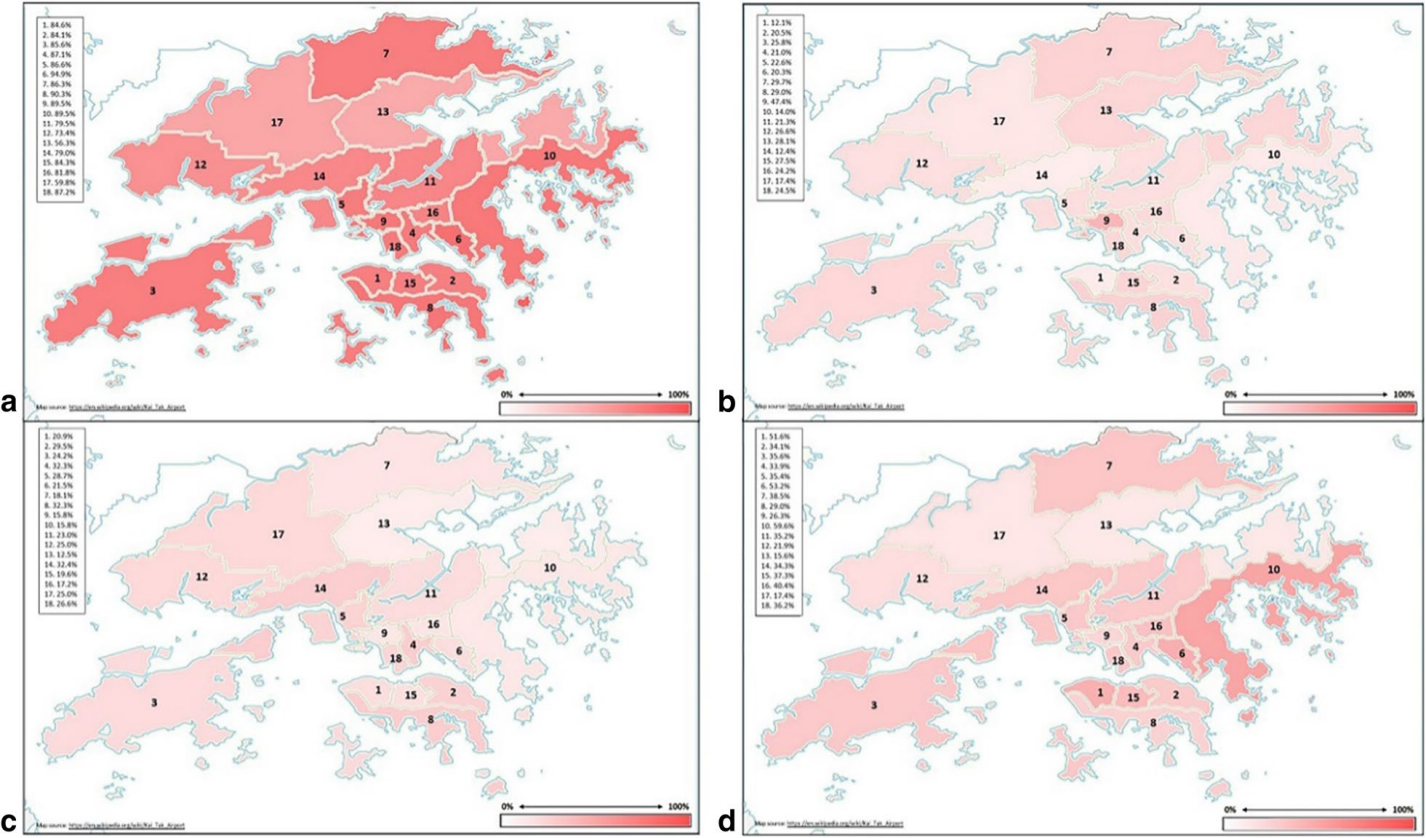
Geographical distribution of *Wolbachia* infection in *Ae. albopictus*. **a** Total infection rate. **b***w*AlbA infection rate. **c***w*AlbB infection rate. **d** Infection rate with both *w*AlbA and *w*AlbB. Key to districts: 1, Central and West: 2, Eastern; 3, Islands; 4, Kowloon City; 5, Kwai Tsing; 6, Kwun Tong; 7, North; 8, Southern; 9, Sham Shui Po; 10, Sai Kung; 11, Sha Tin; 12, Tuen Mun; 13, Tai Po; 14, Tsuen Wan; 15, Wan Chai; 16, Wong Tai Sin; 17, Yuen Long; 18, Yau Tsim Mong

The geographical distribution of *Wolbachia* infection in Hong Kong mosquitoes varied across the territory’s 18 districts (Fig. 2). The area with the highest *Wolbachia* infection rate was the urban district of Kwun Tong in Kowloon (District number 6). 94.9% of mosquito samples collected from Kwun Tong were *Wolbachia*-infected, compared with only 56.3% of the samples collected from the New Territories district of Tai Po (District number 13).

**Fig. 3:**
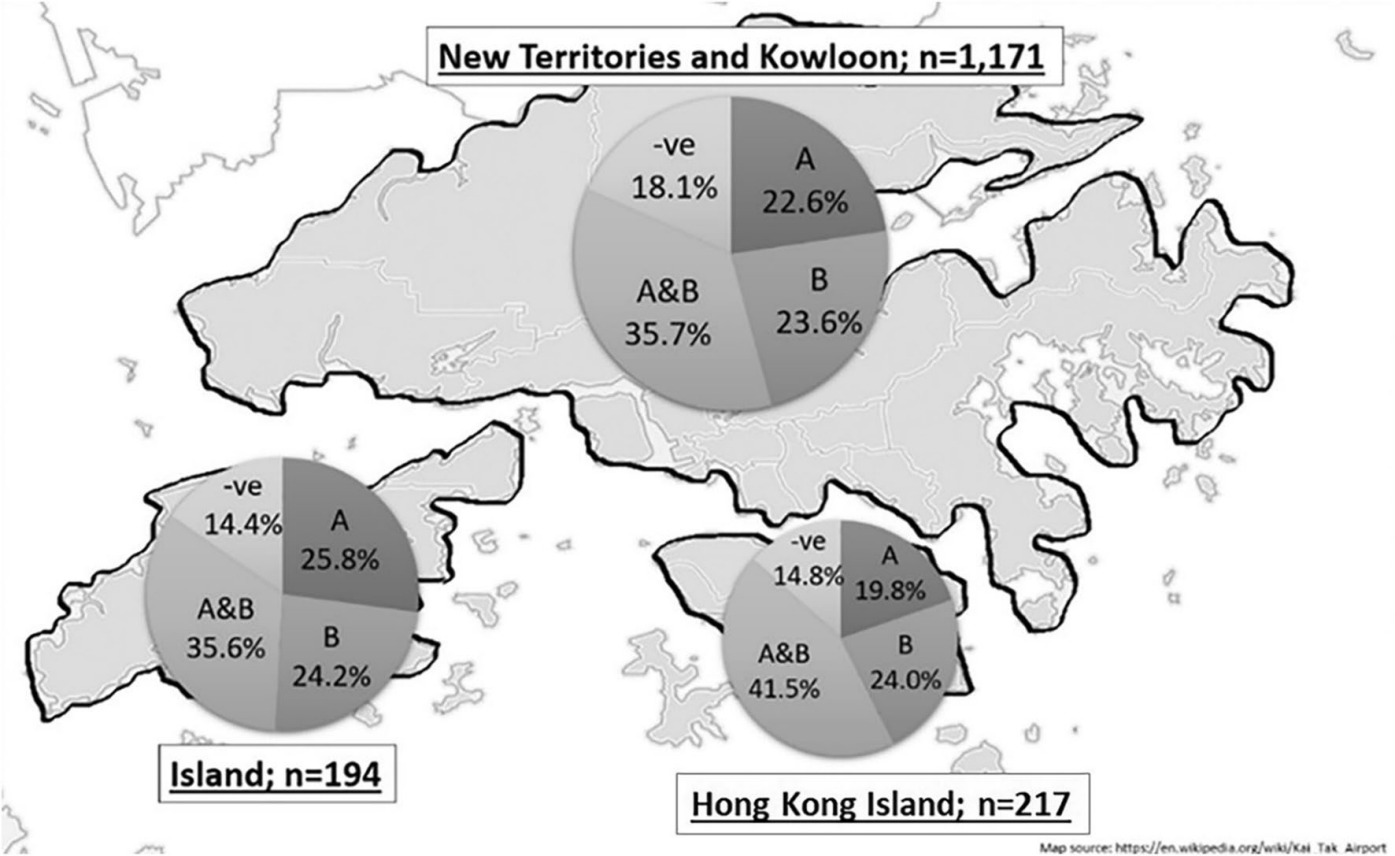
Spatial distribution of *Wolbachia w*AlbA and *w*AlbB infection rates in *Ae. albopictus* in Hong Kong

Overall, the infection rate of either *w*AlbA or *w*AlbB ranged between 12–47.4%, while the infection rate among mosquitoes doubly infected with both *w*AlbA and *w*AlbB was between 14.6–58.9%. In the Chi-square analyses, it was also found that the frequency of infection differed significantly among the 18 districts (*χ*^2^ = 74.5, *df* = 17, *P* < 0.05). As most mosquitoes can fly some distance away from their breeding sites, widening the potential area of infection, the data are also presented according to three broader geographical divisions: (i) the New Territories and Kowloon; (ii) the Outlying Islands; and (iii) Hong Kong Island. These three areas are separated by bodies of seawater (Fig. 3).

## Discussion

*Aedes albopictus* has long been known to be a vector of dengue and Zika viruses in many parts of Southeast Asia. The mosquito can be infected either with single (wAlbA or wAlbB) or multiple (wAlbA and wAlbB) strains of *Wolbachia*. This study reveals the infectious status of dengue and Zika viruses, and *Wolbachia* in mosquitoes in Hong Kong, and reveals markedly different dynamics to those reported previously in other parts of Asia.

The current measures for mosquito control in Hong Kong were established by the HKSAR Government in 2004, when a surveillance programme using ovitraps to monitor the distribution of *Ae. albopictus* in different areas was introduced. As the female mosquito lays her eggs near (as opposed to directly in) water, and has a short flight range (less than two hundred metres), the ovitraps used in this study should provide a representative sample of mosquito distribution across Hong Kong’s various districts at the time of the study.

Our results document a high infection rate (> 80%) of *Wolbachia* in *Ae. albopictus* in Hong Kong. This finding concurs with other published data showing the general trend of natural infection of *Wolbachia* in *Ae. albopictus* [18, 20, 23, 38]. It contrasts markedly with the low infection rate (25%, 71 out of 284 samples) documented in a recent study conducted in Malaysia [35], but is in line with the higher rate found by other studies (94.8%, 271 out of 286 samples) carried out both in Malaysia [39] and in Thailand (100%, 1081 out of 1081 samples) [23].

The infection rate of the dengue virus in Malaysia was also investigated in Teo’s study [35], and was found to be 25.7% (73 out of 284 samples). By contrast, our own study found no evidence for the existence of dengue virus in Hong Kong (0 out of 967 samples).

While the study by Joanne et al. [39] in Malaysia found a high *Wolbachia* infection rate in *Ae. albopictus*, 91.6% of its samples were superinfected with both wAlbA and wAlbB, and very few were singly infected (~ 1% with wAlbA and ~ 2% with wAlbB). A similar pattern has also been documented in Thailand, where 99.41% of samples were doubly infected with both *Wolbachia* strains) [23]. Although the rate of double infection was also found to be higher than that of single infection in all eighteen districts in Hong Kong, around 40% of our own mosquito samples were singly infected. Assuming the observed differences are not built on the different methodologies employed in different studies, these data suggest that the composition of *Wolbachia* could be different in *Ae. albopictus* in different parts of Southeast Asia. This phenomenon is comparable to the pattern of *Wolbachia* infection in tsetse flies, where infection rates vary from region to region [40].

The dynamics of *Wolbachia* composition remain relatively constant during mosquito’s breeding season (April to October) in Hong Kong. The striking local *Wolbachia* infection pattern of *Ae. albopictus* in Hong Kong (~ 47% of samples singly-infected, and ~ 36.8% doubly infected with wAlbA and wAlbB) remained relatively stable over the study period. Further studies in a few years’ time would enable us to verify whether this composition has undergone any change.

Detailed statistical analyses for *Wolbachia* infection were also carried out and shown in Additional file 1: Tables S5–S8. Differences were found between the infection rates in Kowloon and New Territories to the islands. In particular, mosquitoes in District numbers 6, 13 and 17 (Kwun Tong, Tai Po and Yuen Long) have different infection rates than expected values. Considering that 64 to 92 mosquitoes were taken from each of these three districts, future follow-up work will also be required to determine whether this is an artifact of sample size, or whether this represents the human population/ecology of these districts.

In the past few decades, vector control methods have become heavily dependent on the use of insecticides, and resistance to insecticides in *Ae. albopictus* is increasingly giving grounds for concern [41]. Given the cytoplasmic incompatibility caused by *Wolbachia* bacteria on *Aedes* mosquitoes, elimination programs using *Wolbachia*-infected mosquitoes to replace natural mosquito populations have been carried out in several countries, including Australia, Brazil, Colombia, Indonesia and Vietnam [15]. In some parts of Asia, programs have also been developed which involve the release of genetically modified mosquitoes.

Against this background, it is worth considering whether a case exists for the introduction of alternative mosquito control methods in Hong Kong. It is not yet clear whether the zero infection rate of both dengue and Zika viruses in *Ae. albopictus* in Hong Kong reflects the consequences of *Wolbachia* infection, the effectiveness of the government’s program for the regular application of insecticides, changes in the genetics of the mosquito population, or a combination of these causes. Alternatively, these viruses may be present in the local mosquito population at such low levels that they cannot easily be detected in infected individuals. However, the latter possibility seems unlikely, as the complete absence of evidence for the existence of dengue virus in Hong Kong (0 out of 967 samples) contrasts markedly with the prevalence of infection found in one of the Malaysian studies (73 out of 284 samples) [35]. In any case, given that very few cases of infection with either dengue or Zika virus have occurred in Hong Kong, there is no compelling case at present for introducing alternative control methods. Nevertheless, it would be prudent to conduct further investigations to improve our knowledge of infection patterns in local mosquitoes, so that our existing control methods can be reviewed, if necessary, in the light of our growing scientific knowledge.

## Conclusions

The study sheds important light on the pattern of disease-causing agents and endosymbionts in *Ae. albopictus* in Hong Kong over a study period that lasted just over one year. The study reveals a distinctive pattern of infection that differs in several respects to those found elsewhere in Asia.

## Supplementary information

**Additional file 1: Table S1.** Sample information used in the Zika test. **Table S2.** Sample information used in the dengue test. **Table S3.** Sample information used in the *Wolbachia* test. **Table S4.** a Monthly number of tested samples between 2018 and 2019. b–f Data summary of Table S1–S3. **Table S5.** Chi-square analyses of total infection frequency between three sites. **Table S6.** Chi-square analyses of different *Wolbachia* infection types between New Territories & Kowloon and other sites. **Table S7.** Chi-square analyses of total infection frequency between 18 districts. **Table S8.** Chi-square analyses of different *Wolbachia* infection types between four districts.

**Additional file 2: Figure S1.***Wolbachia* infection in April 2018. **Figure S2.***Wolbachia* infection in May 2018. **Figure S3.***Wolbachia* infection in June 2018. **Figure S4.***Wolbachia* infection in July 2018. **Figure S5.***Wolbachia* infection in August 2018. **Figure S6.***Wolbachia* infection in September 2018. **Figure S7.***Wolbachia* infection in October 2018. **Figure S8.***Wolbachia* infection in November 2018. **Figure S9.***Wolbachia* infection in December 2018. **Figure S10.***Wolbachia* infection in February 2019. **Figure S11.***Wolbachia* infection in March 2019. **Figure S12.***Wolbachia* infection in April 2019.

## Data Availability

Not applicable.

## Abbreviations

CI: cytoplasmic incompatibility
ovitrap: oviposition traps
PCR: polymerase chain reaction
