# Supplementary material for: Infection patterns of dengue, Zika and endosymbiont Wolbachia in the mosquito Aedes albopictus in Hong Kong
Source: Parasit Vectors. 2020 Jul 20;13:361. doi: 10.1186/s13071-020-04231-x (PMC7372788; doi:10.1186/s13071-020-04231-x)
Supplement: Supplementary file 2 — Additional file 2: Figure S1.Wolbachia infection in April 2018. Figure S2.Wolbachia infection in May 2018. Figure S3.Wolbachia infection in June 2018. Figure S4.Wolbachia infection in July 2018. Figure S5.Wolbachia infection in August 2018. Figure S6.Wolbachia infection in September 2018. Figure S7.Wolbachia infection in October 2018. Figure S8.Wolbachia infection in November 2018. Figure S9.Wolbachia infection in December 2018. Figure S10.Wolbachia infection in February 2019. Figure S11.Wolbachia infection in March 2019. Figure S12.Wolbachia infection in April 2019. [file 13071_2020_4231_MOESM2_ESM.docx]

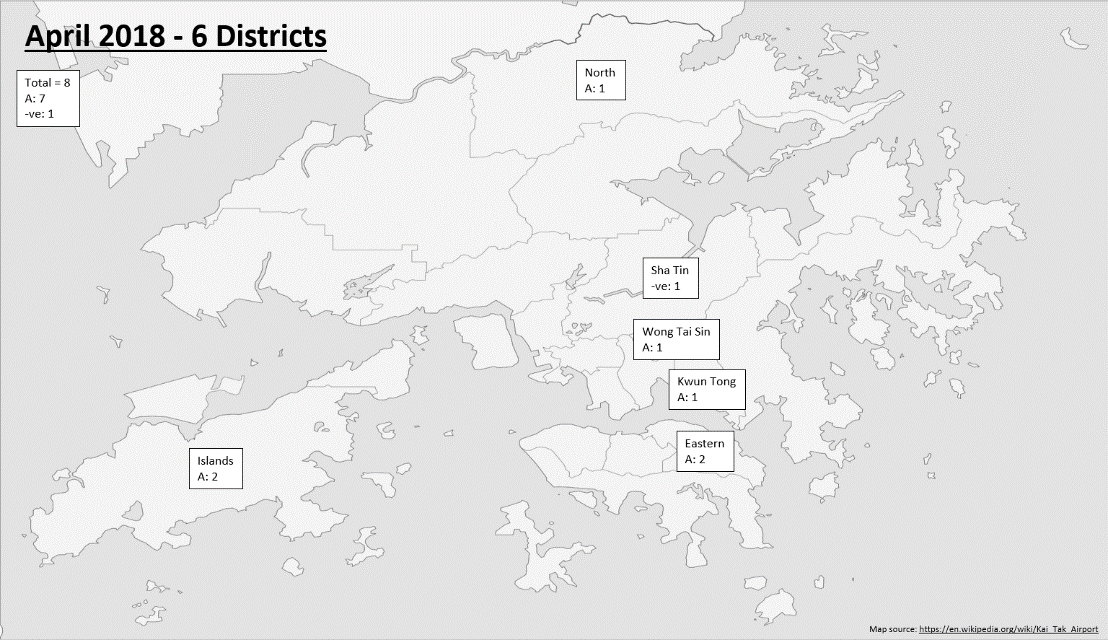


Figure S1. *Wolbachia* Infection in April 2018


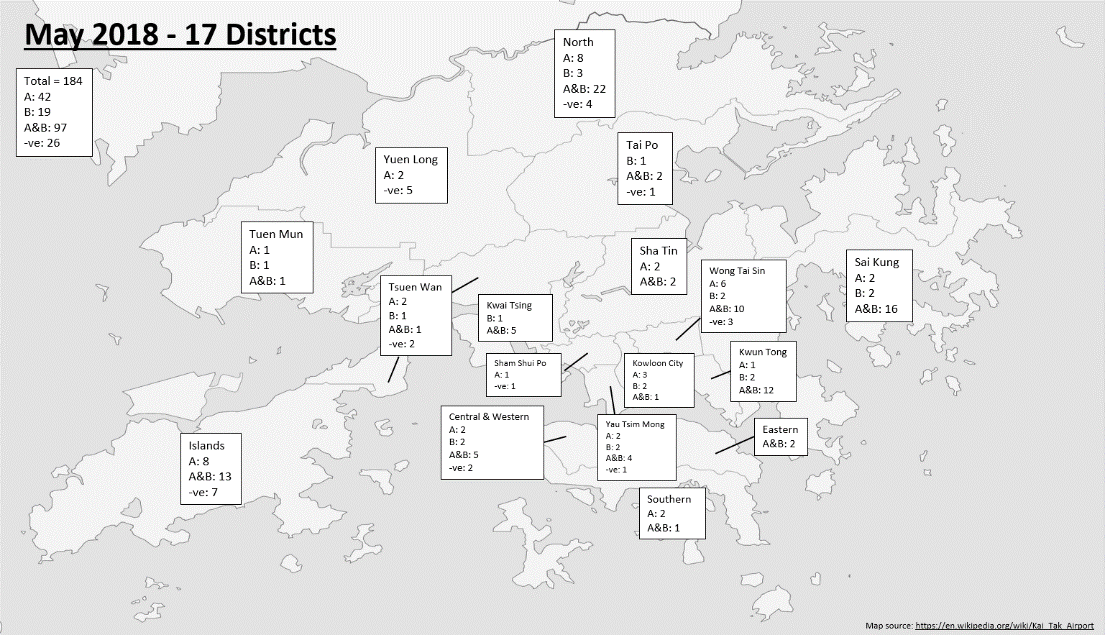


Figure S2. *Wolbachia* Infection in May 2018


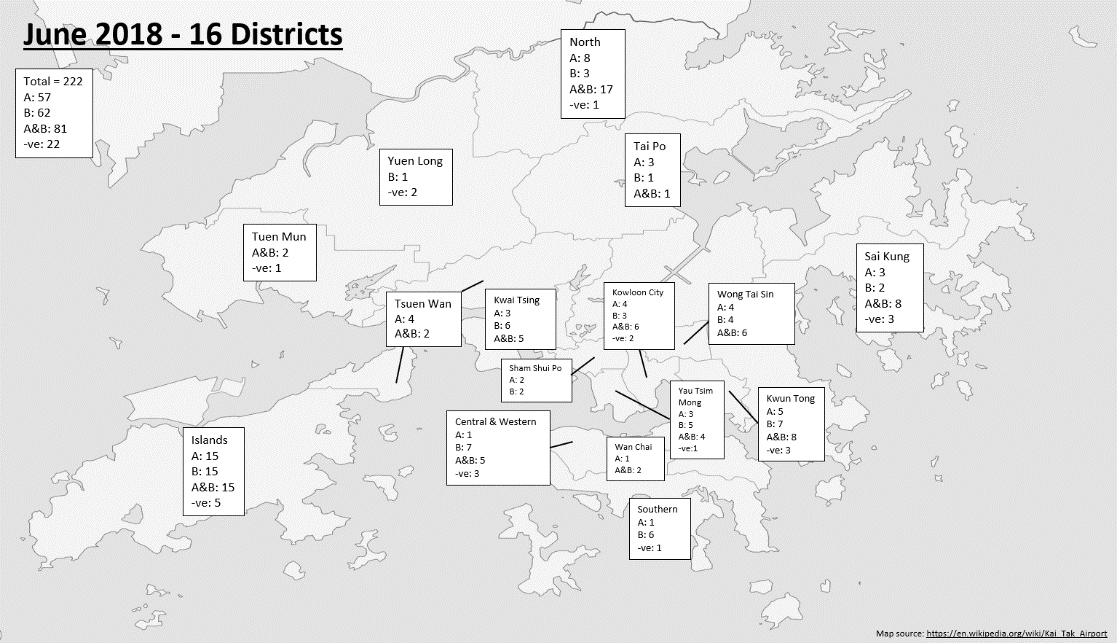


Figure S3. *Wolbachia* Infection in June 2018


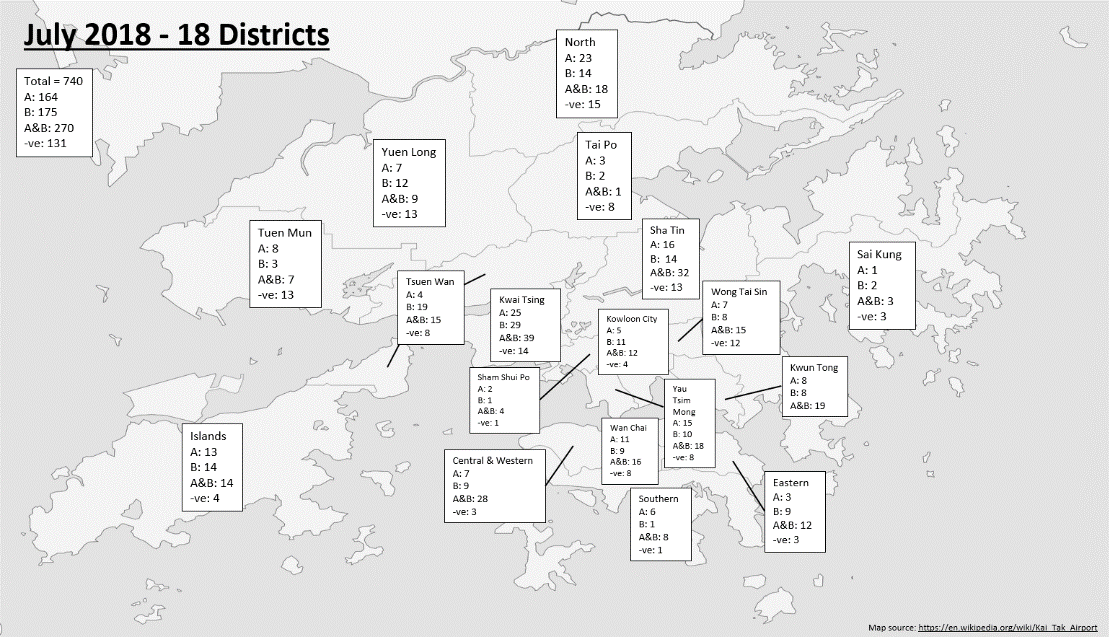


Figure S4. *Wolbachia* Infection in July 2018


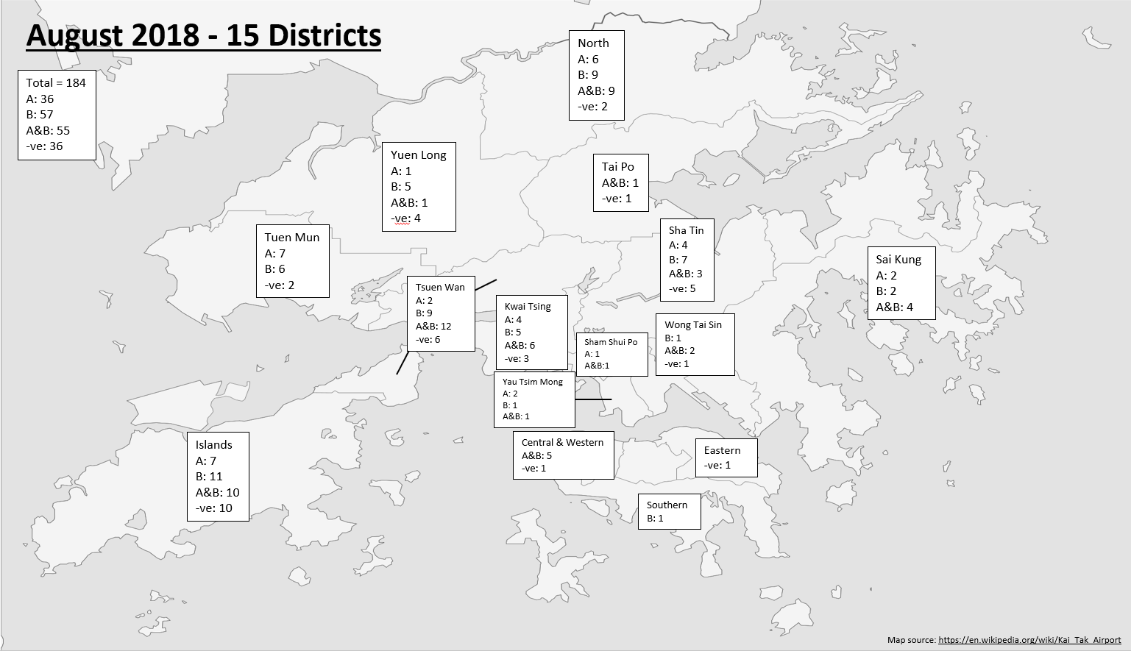


Figure S5. *Wolbachia* Infection in August 2018


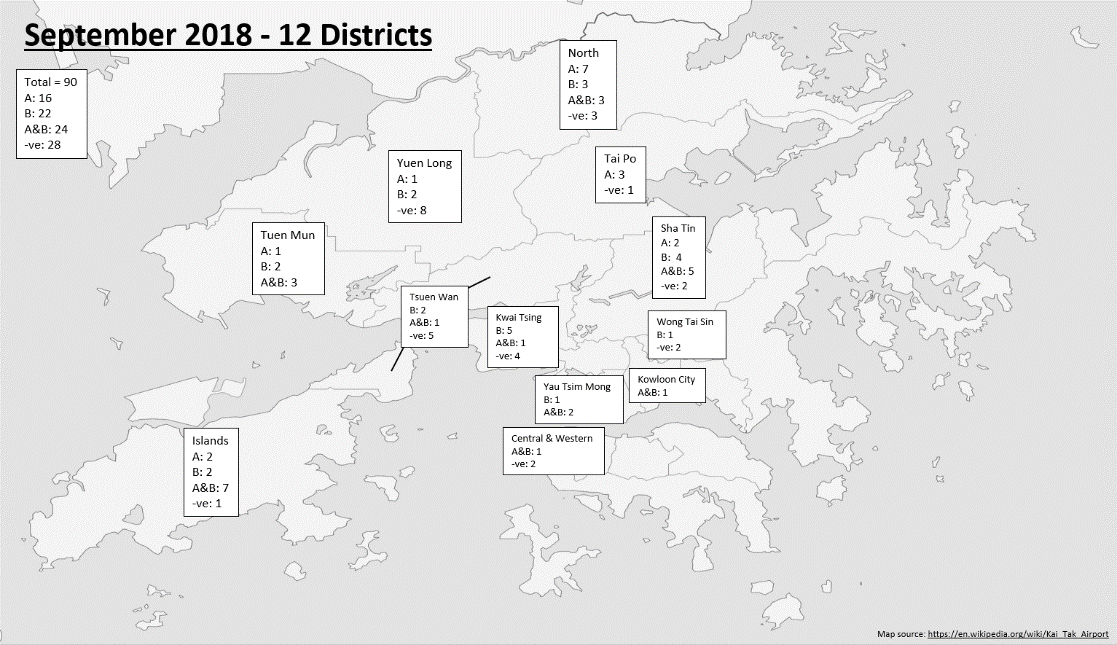


Figure S6. *Wolbachia* Infection in September 2018


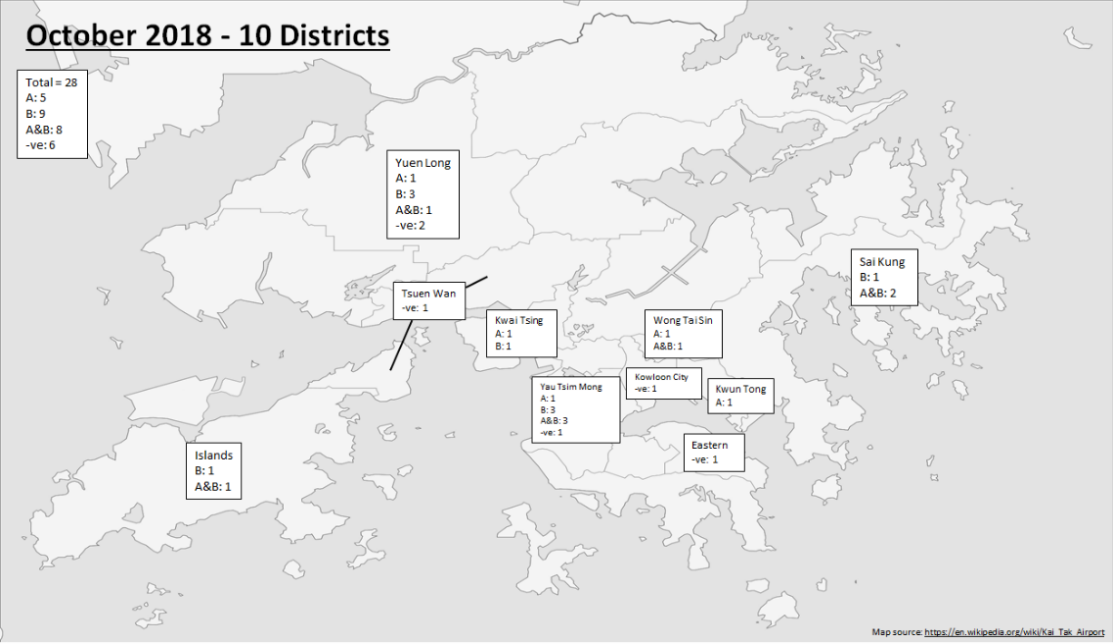


Figure S7. *Wolbachia* Infection in October 2018


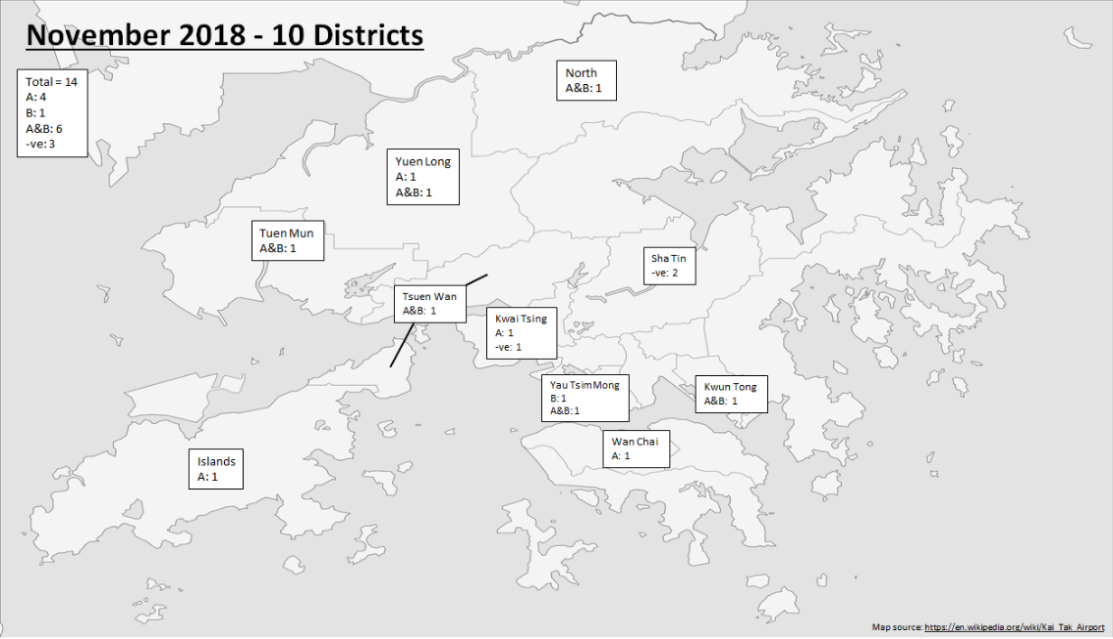


Figure S8. *Wolbachia* Infection in November 2018


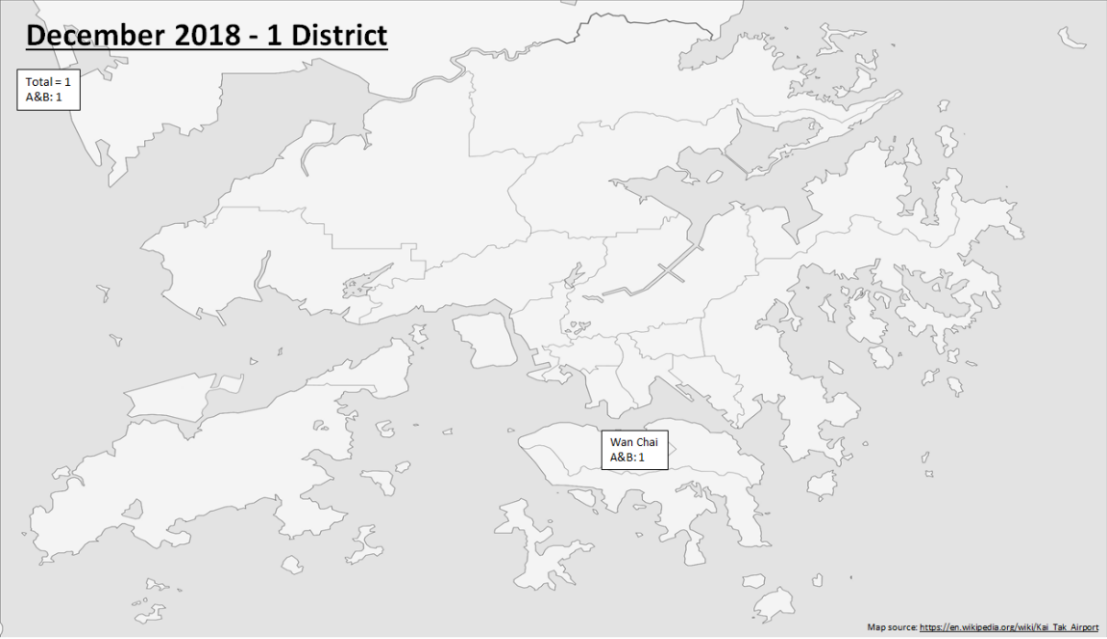


Figure S9. *Wolbachia* Infection in December 2018


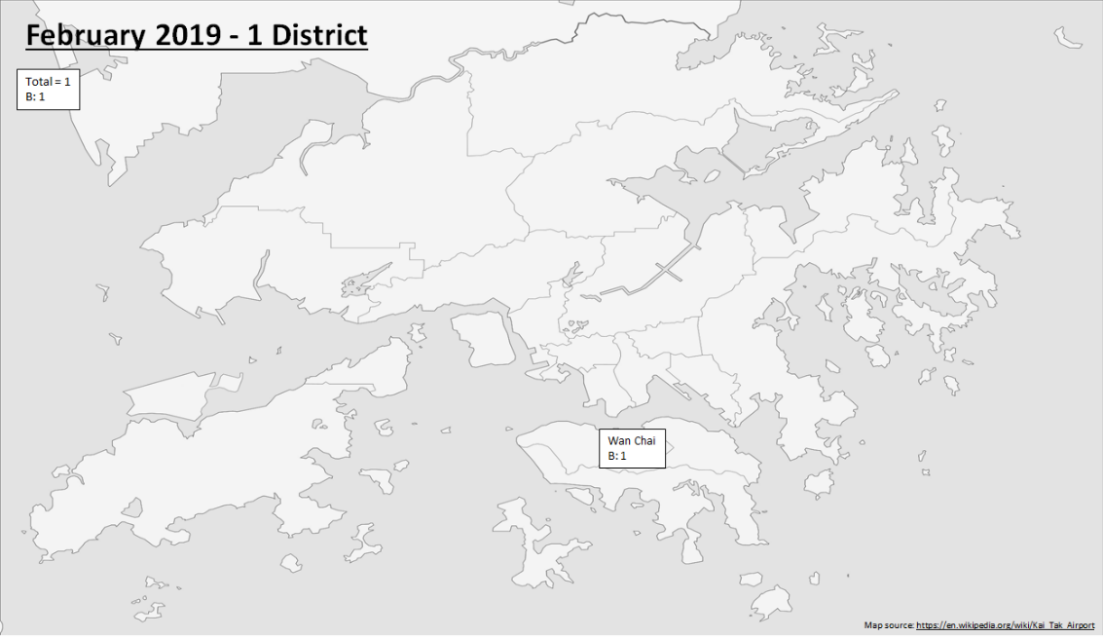


Figure S10. *Wolbachia* Infection in February 2019


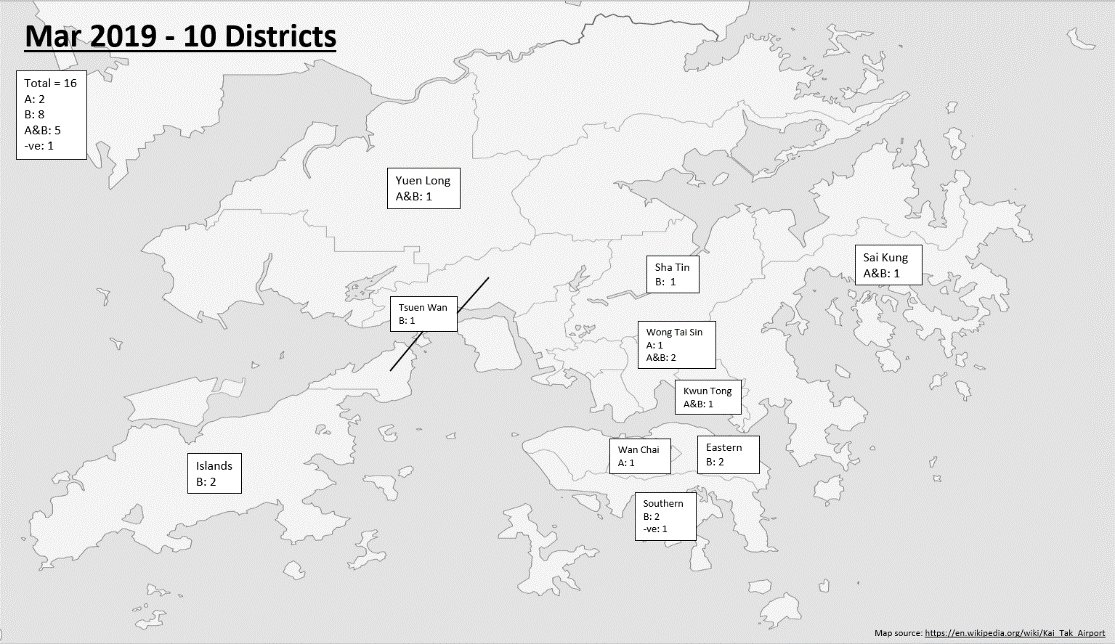


Figure S11. *Wolbachia* Infection in March 2019


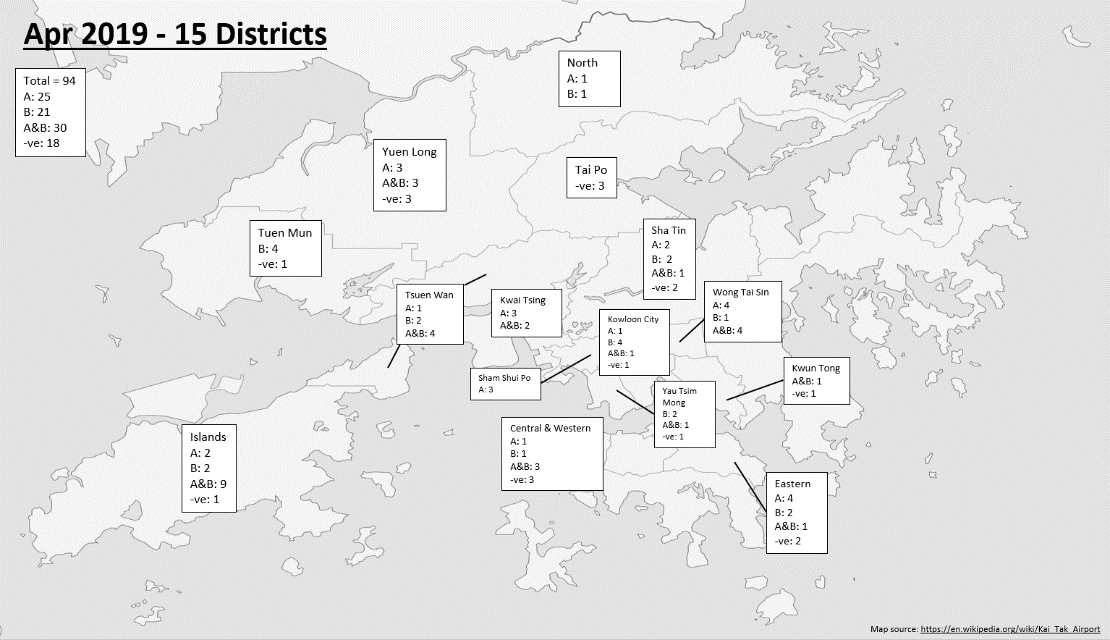


Figure S12. *Wolbachia* Infection in April 2019
